# Electronic tools to improve procalcitonin utilization

**DOI:** 10.1017/ash.2024.501

**Published:** 2025-02-11

**Authors:** Julia A. Hisey, Grace K. Mahowald, Nicole V. Tolan, Phillip Kang, Ramy H. Elshaboury, Anand S. Dighe, Kent B. Lewandrowski, Christiana A. Demetriou, Stacy E.F. Melanson, Alyssa R. Letourneau

**Affiliations:** 1 Tufts School of Medicine, Boston, MA, USA; 2 Department of Pathology, Mass General Brigham, Boston, MA, USA; 3 Harvard Medical School, Boston, MA, USA; 4 Department of Pharmacy, Massachusetts General Hospital, Boston, MA, USA; 5 Department of Primary Care and Population Health, University of Nicosia Medical School, Nicosia, Cyprus; 6 The Cyprus School of Molecular Medicine, The Cyprus Institute of Neurology and Genetics, Nicosia, Cyprus; 7 Department of Medicine, Division of Infectious Division, Massachusetts General Hospital, Boston, MA, USA

## Introduction

Procalcitonin (PCT) is a biomarker of bacterial infections and used for antimicrobial stewardship.^1^ Clinical trials support PCT to: (1) rule out bacterial lower respiratory tract infections in non-critically ill patients, (2) rule out bacterial sepsis in critically ill patients, and (3) deescalate antibiotics.^1^ Studies have demonstrated inappropriate utilization of PCT, a high-cost test, including frequent retesting or failing to discontinue antibiotics.^2^–^4^ To improve appropriate utilization, institutions have altered order sets, audited PCT ordering, and provided education, all with varying effects.^2^,^3^,^5^ Our institution recently standardized PCT ordering and changed our reference range from ≤0.08 to ≤0.25 so that flagged/abnormal results reflect local guidelines. Here, we assess the impact of electronic tools to decrease unnecessary PCT orders.

## Methods

### Study settings

This study was conducted at Brigham and Women’s Hospital (BWH) and Massachusetts General Hospital (MGH), two large tertiary care centers in Boston, MA. BWH implemented two tools in the electronic health record (EHR) (Epic Systems Corporation, Madison, WI).

### Interventions

First, at the time of ordering, the clinician was presented with prior results, if any, order education (ie PCT is not indicated in the following situations: known chronic bacterial infection requiring long-term antibiotics, severely immunocompromised (other than corticosteroids), or acute COPD exacerbation) AND was required to indicate their reason for ordering PCT (ie 1-suspected bacterial respiratory infection in a non-critically ill patient, 2-suspected sepsis in a critically ill patient, 3-repeat test within 24 hours: initial result ≤ 0.25 and high suspicion for respiratory infection or sepsis, 4-repeat test within 24 hours: symptoms persist or are worsening, 5-other (enter comment)) (Supplemental Figure 1). Second, a duplicate checking reminder was displayed if a PCT resulted in the past 48 hours; the previous result was displayed and the option to cancel was given (Supplemental Figure 2).

The intervention was implemented at BWH on January 23, 2024. Pre-intervention was February 1, 2022 to December 31, 2023 and post-intervention was February 1, 2024 to June 30, 2024. We determined normalized PCT volumes pre- and post-intervention at BWH and MGH (where PCT ordering was already restricted), calculated the number of PCT results per patient per encounter pre- and post-intervention at BWH and MGH, monitored the impact of duplicate checking reminder at BWH, and reviewed medical records at BWH post-intervention to assess the accuracy of responses to order questions (n = 50 medical records, 10 for each response).

### Statistical analysis

Joinpoint regression analysis^6^ was used (SEER*Stat software (Version 4.9.0.0)) to estimate the monthly percentage change (MPC) in normalized PCT volumes. In this analysis, the MPC was calculated by fitting a linear regression model on log-transformed trends, using month as the independent variable, under the assumption of constant variance and uncorrelated errors. A maximum of five change points were allowed. Where a changing trend was detected, each trend line segment was expressed by an MPC value. The number of PCT results per patient per encounter was compared pre- and post-intervention using negative binomial regression and **Stata v.18 software (StataCorp)** to account for over-dispersion of data points.

## Results

**Figure 1.**
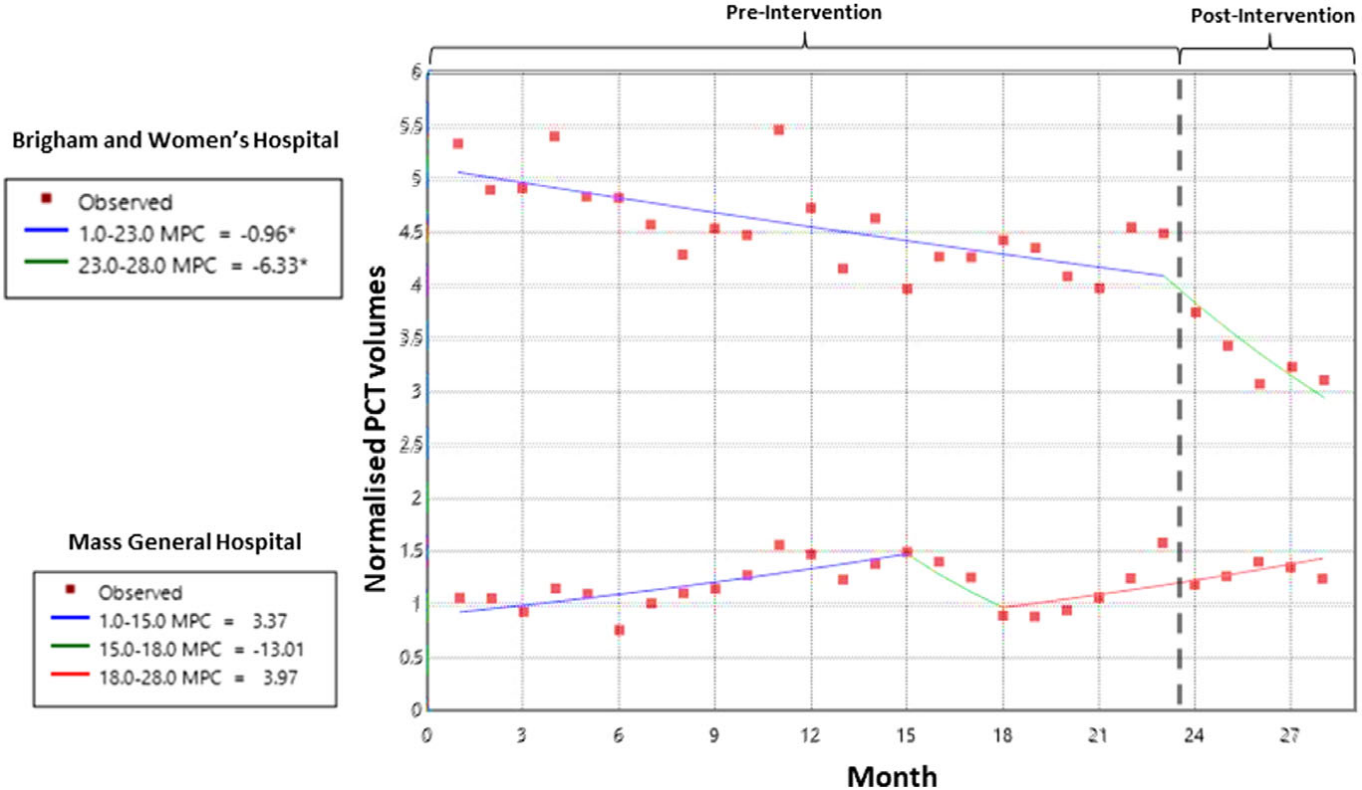
Joinpoint regression plots for Brigham and Women’s Hospital (top half) and Massachusetts General Hospital (bottom half) from January 2022 to June 2024. Normalized monthly procalcitonin volumes are plotted. Differential trends in monthly percent changes are shown by a different color line. An asterisk indicates a significant trend change.

PCT orders post-intervention decreased significantly at BWH (MPC post vs pre: -6.33% vs -0.96%) but not at MGH (MPC: 3.97%) (Figure 1). The average number of PCT tests ordered per patient per encounter decreased significantly during the post-intervention period at BWH (negative binominal regression incidence rate ratio (IRR) of 0.87, 95% Confidence Interval (CI): 0.83–0.90, *P* < 0.001), while there was no significant change at **MGH (IRR of 0.97**, 95% CI: 0.92–1.02, *P* = 0.23). At BWH, 54.5% (1,373), 27.6% (695), 2.0% (50), 1.9% (49), and 14.0% (354) of clinicians chose option 1, 2, 3, 4, or 5; respectively (n = 2,521). Of the “other” responses (option 5), 80.2% (284/354) were to distinguish bacterial infection from cytokine release syndrome in CAR-T cell patients. As determined by patient location in EHR, PCT was ordered if suspected bacterial respiratory infection in a non-critically ill patient (208 of 955; 21.7%) or suspected sepsis in a critically ill patient (210 of 625; 33.6%). Further, in 31 of the 50 (62%) patients reviewed, PCT was inconsistent with the order education and not clinically indicated.

## Discussion

We demonstrated that electronic tools in the EHR can significantly decrease PCT ordering. Given the success at BWH, we have implemented these tools across our hospital system and will monitor the system-wide impact and associated PCT reagent cost savings. Despite the decrease in volume, PCT is still ordered when not indicated, such as in severely immunocompromised patients. The majority of clinicians who proceed with ordering PCT are selecting option 1, even in critically ill patients, suggesting that selection is not based on the patient’s history but that they are simply clicking a button to expedite ordering. Further, we identified that PCT was commonly ordered in CAR-T cell recipients. To address these limitations, we will discuss utilization in CAR-T cell patients with clinical leaders and follow up with individual clinicians who are ordering PCT when not clinically indicated.

## Supporting information

Hisey et al. supplementary material 1 Hisey et al. supplementary material

Hisey et al. supplementary material 2 Hisey et al. supplementary material

## Supplementary material

The supplementary material for this article can be found at https://doi.org/10.1017/ash.2024.501
