# Supplementary material for: Electronic tools to improve procalcitonin utilization
Source: Antimicrob Steward Healthc Epidemiol. 2025 Feb 11;5(1):e33. doi: 10.1017/ash.2024.501 (PMC11822617; doi:10.1017/ash.2024.501)
Supplement: Hisey et al. supplementary material 1 — Hisey et al. supplementary material [file S2732494X24005011sup001.docx]

**Supplemental Figure Legends**

**Supplemental Figure 1.** The guidance provided to clinicians when placing an order for procalcitonin is shown, with each element of the guidance numbered and outlined in a blue box. Clinicians are asked to choose the clinical indication for testing (1) and are presented with process instructions which detail the situations in which procalcitonin testing is not necessary (4). Previous results, if any, are displayed at the time of ordering (2). Reference links to both the laboratory handbook and the MGB procalcitonin guidelines are available (3).

**Supplemental Figure 2.** The duplicate check reminder that displays when a clinician orders a procalcitonin test within 48 hours of a previous order is shown. The most recent result together with the specimen date/time is presented (blue box). The provider has the option to continue or cancel the order.
