# Supplementary figures and images for: Electronic tools to improve procalcitonin utilization
Source: Antimicrob Steward Healthc Epidemiol. 2025 Feb 11;5(1):e33. doi: 10.1017/ash.2024.501 (PMC11822617; doi:10.1017/ash.2024.501)

## Slide 1
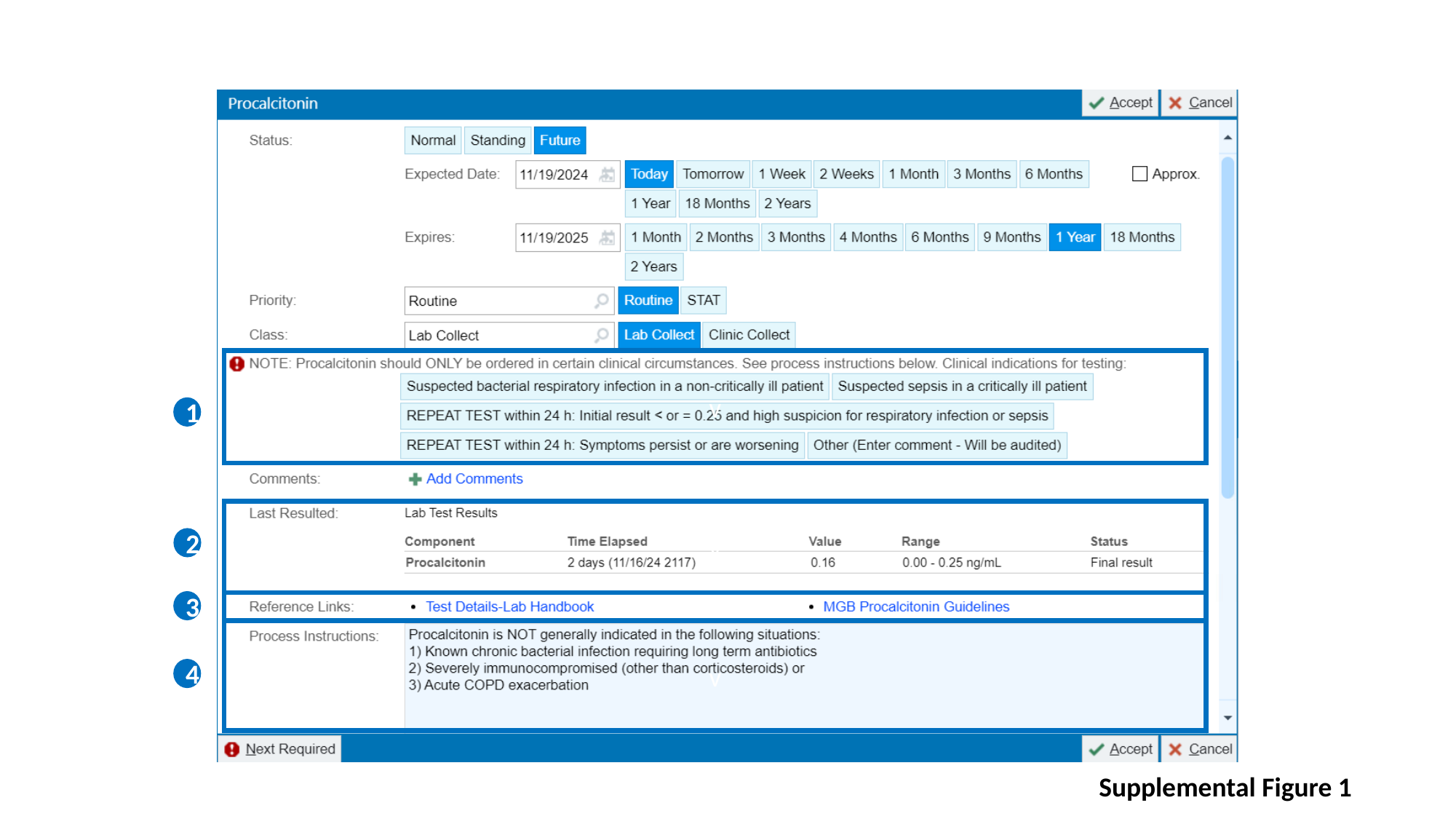

v
1
v
2
3
v
4
Supplemental Figure 1

## Slide 2
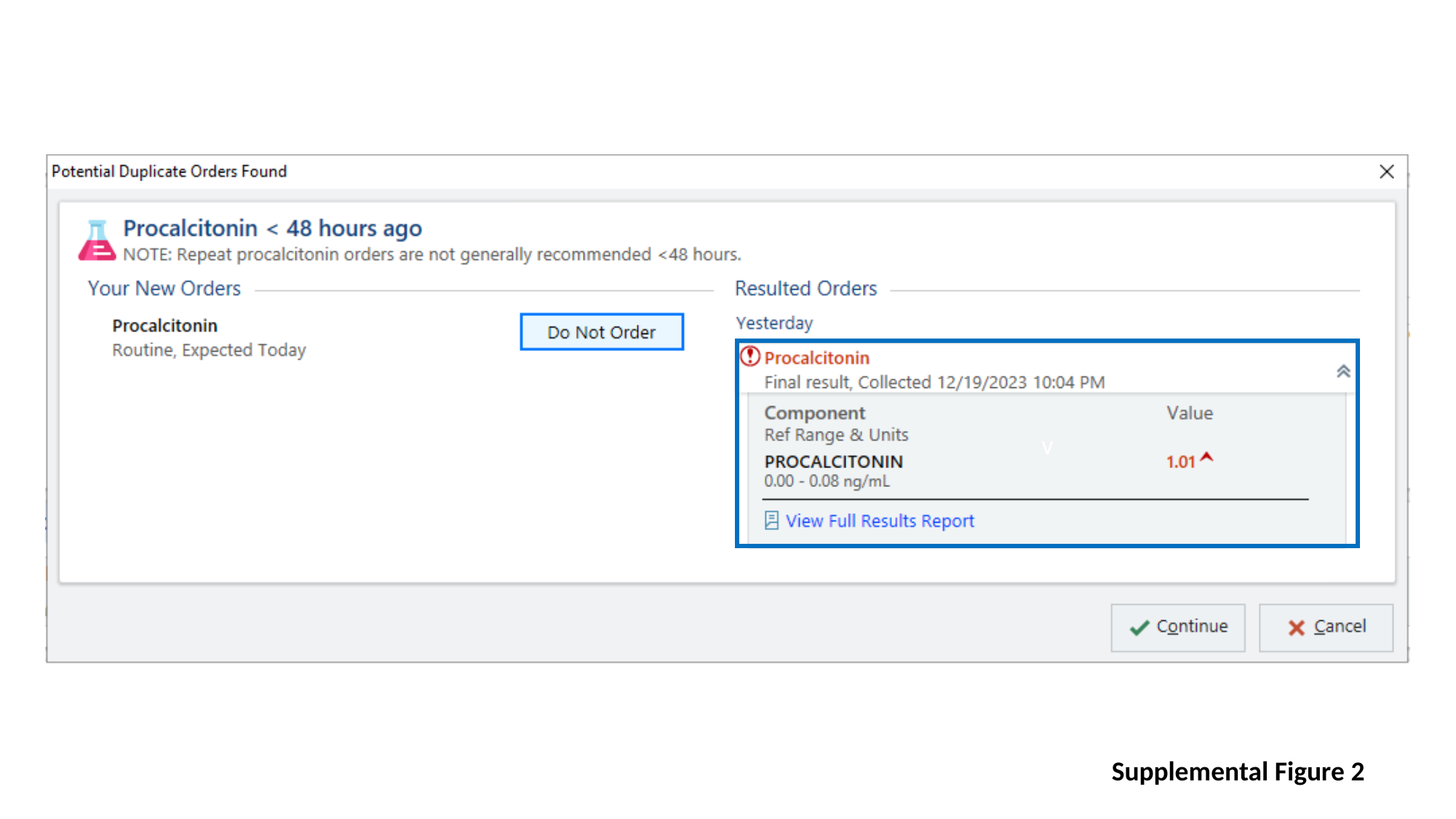

v
Supplemental Figure 2

Supplement: Hisey et al. supplementary material 2 — Hisey et al. supplementary material [file S2732494X24005011sup002.pptx]
